# Improving the thermal tolerance of biocontrol spores, *Akanthomyces lecanii*, by encapsulation

**DOI:** 10.1093/femsle/fnaf062

**Published:** 2025-06-16

**Authors:** Paul W Baker, Ephraim Nuwamanya, Adam Charlton

**Affiliations:** Biocomposites Centre, Bangor University, Deiniol Road, Bangor, Gwynedd LL57 2UW, Wales, United Kingdom; College of Agricultural and Environmental Sciences, Makerere University, University Road, 7062 Kampala, Uganda; Biocomposites Centre, Bangor University, Deiniol Road, Bangor, Gwynedd LL57 2UW, Wales, United Kingdom

**Keywords:** improving, thermal, tolerances, biocontrol, spores, *Akanthomyces*

## Abstract

*Akanthomyces lecanii* is an entomopathogenic fungus, and spores of this fungus could be incorporated into films generated using cast film extrusion for biocontrol applications. However, the extrusion process involves high temperature processing (150°C) although this only lasts for a few minutes. The elevated temperature destroys spores, thereby eliminating functionality, unless the spores are protected from this heat. Initial experiments revealed that the heat tolerance of free *A. lecanii* spores to be 60°C. The spores were therefore encapsulated into beads prepared using a combination of Gelrite, cellulose, and Cel-fine at different concentrations. The beads were freeze-dried and then immersed in hot glycerol for 2 min at a selected temperature within the range of 50°C–100°C. The results indicated that some combinations of encapsulating agents resulted in the spores retaining viability (plate counting) after heat treatment at 100°C. Beads stored at room temperature for 1 week showed a reduction in the upper temperature tolerance. This study revealed that the temperature tolerance of *A. lecanii* spores could be improved by 40°C by encapsulation in freeze-dried beads containing 2% Gelrite (purified gellum gum), 0.4% cellulose, and 0.4% Cel-fine.

## Teaser

Improving the heat tolerance of an entomopathogenic fungus by encapsulation for use in extrusion.

## Introduction

In recent years, encapsulation technology has been developed for the use in agriculture by providing a slow release of plant extracts that have insecticidal activity (Pinto et al. 2022) or in the release of essential nutrients required by the plant or to prevent desiccation of biostimulating microorganisms (Wang et al. 2023). Many years of research have investigated different suspensions used to encapsulate bacteria, fungi, plant cells, and animal cells often with the purpose of producing metabolites and enzymes (Willaert and Baron 1996). Besides the rapid progress in production of chemicals by encapsulated microbes, this technology has increasing application in the probiotic industry to protect the microorganisms from harmful digestive juices in the gut (de Souza et al. 2022).

**Figure 1.**
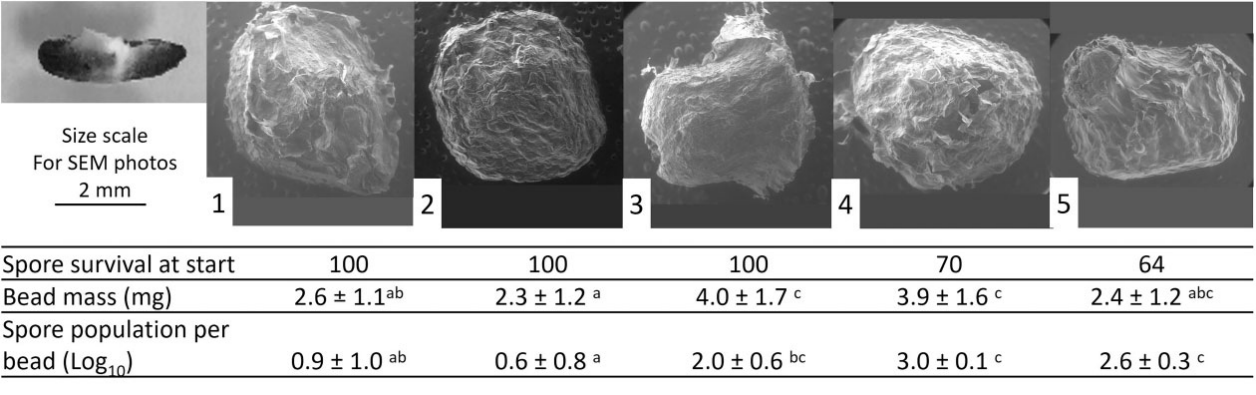
Top right: mycelia of *A. lecanii* appeared from the dead body of a waxworm. Scanning electron microscopy photos of beads prepared with suspensions labeled as 1–5 (see Table 1 for composition). The table below shows the averages of bead masses (*n* = 15), calculated diameters from density measurements (*n* = 15), and spore population associated with each bead (*n* = 5) to the corresponding photos above. Values with the same letters are not significantly different from each other and values with different letters are significantly different (*P* < .05).

There are 750 different species of fungi that can infect insects, which are morphologically and phylogenetically different from one another (Islam et al. 2021). The specialized entomopathogenic fungi invade the body cavity through the exoskeleton or through the mandible region leading to death of the infected insect, and mycelia appear from the surface of the corpse, if humidity conditions are sufficiently high (Fig. 1). Such effects were observed in a previous study where 10^5^ and 10^9^ spores of *Metarhizium brunneum* correlated with the appearance of mycelia from 33% and 100% of the corpses, respectively (Yousef et al. 2014). One commonly described entomopathogenic fungus, *Beauveria bassiana* can infect a wide range of insects (Mascarin and Jaronski 2016) and has been used in the biological control of the two-spotted spider mites (*Tetranychus urticae*), known to cause significant damage to vegetable crops (de Oliveira et al. 2023). In this study, the spores of *B. bassiana* were encapsulated in order to improve the survival of these spores in the natural environment. The use of encapsulation specifically for biocontrol fungi as insecticides has gradually been generating interest (Mascarin and Jaronski 2016). Different formulations were examined although maltodextrin/gum Arabic was found to be optimal when used in combination with spray drying at 100°C. The use of spray drying resulted in the formation of smaller diameter particles measuring 23.5 µm. In another study, a comparison was made between ionic gelation and spray drying for the encapsulation of *B. bassiana* using a variety of different additional reagents such as cellulose, soya oil, humic acids, lignin, and sodium alginate in a factorial combination (Felizatti et al. 2021). This study reported that many of the formulation parameters investigated by factorial design in terms of air inlet speed, inlet temperature (70°C–120°C), feed flow rate and aspirator rate showed that under some conditions the encapsulated spores had an improved resistance to UV over 48 h and to heat treatment at 60°C for 4 h compared with the free conidia. *Akanthomyces lecanii* is one of four main biocontrol fungi that has been used in commercial applications and encapsulation is a process that could improve tolerance of these fungi to heat and UV light (Sharma et al. 2023).

*Akanthomyces lecanii*, which has undergone reclassification is the modern synonym for the previous basiosym, *Cephalosporium lecanii*, and the synonyms, *Verticillium lecanii* and *Lecanicillium lecanii* (Kepler et al. 2017). The fungi within *Akanthomyces* form a separate clade to the typical *Cordyceps* and *Beauveria* species. The fungus was originally isolated from mites infecting tomato plants amid the concern that aflatoxins associated with the fungus could be carried over into the tomatoes. The fungus has been demonstrated to have activity within the natural environment mostly on Hemiptera (Kepler et al. 2017, Gopal et al. 2021) and some activity has been described on other insects within the orders: Blattodea, Coleoptera, Orthopteta, and Lepidoptera (Xu et al. 2011). Some experimental studies have shown activity on cockroaches, from the order Blattodea (Davari et al. 2015, Hernández-Ramírez et al. 2020, Er et al. 2022), but few studies have investigated the suborder of Isoptera, which comprises termites (Khan et al. 1991). Most research has been performed with *Metarhizium anisopliae* and *B. bassiana* in the biological control of termites (Rath 2000), which found that at least 100 conidia per termite were required for complete mortality of *Coptotermes formosanus* Shiraki (Wang and Powell 2003). *Beauveria bassiana* was less effective against another termite species, *Reticulitermes flavipes* (Kollar). Strains of *B. bassiana* were found to be virulent against >50% workers and soldiers of the termite, *Odontotermes brunneus*, but a strain of *A. lecanii* was shown to be ineffective (Khan et al. 1991). However, it is possible that the *A. lecanii* spores would be effective against termite nymphs, which would lack the hardened exoskeleton or may act as a deterrent toward termites (Xiong et al. 2019).

An ongoing research program at Bangor University was investigating approaches to develop termite repellent polymer formulations, produced by cast film extrusion. However, the incorporation of biocontrol spores during extrusion would be destroyed by the high temperature, which occurs for a short period during the process. It was proposed to encase the spores within a protective matrix to provide the spores with some resistance to the high temperature. Gelrite, a purified form of gellum gum that forms a double helix in the presence of ions which combine to create layers (Banik et al. 2000), was chosen because it forms a rigid gel along with additional inorganic and high molecular weight substances that could enhance temperature tolerance, stability, and impermeability (Willaert and Baron 1996, de Souza et al. 2022). Therefore, experiments were performed to test whether encapsulation could sufficiently increase the heat tolerance of the spores.

## Materials and methods

### Materials

*Beauveria bassiana* NCPF 7468 was obtained from the National Collection of Pathogenic Fungi, UK and *A. lecanii* CECT 20413 was obtained from the Spanish Type Culture Collection. The encapsulated beads were prepared with Duchefa Gelrite (Melford Laboratory Ltd. UK), microcrystalline cellulose with average particle size of 50 µm (Fisher Scientific, UK) and standard superfine Cel-fine, calcined diatomaceous earth (Merck, UK). Tubes, solutions, and suspensions were autoclaved at 121°C for 15 min. in a benchtop medical autoclave (Prestige, UK) and suspensions containing Gelrite were maintained in a linear shaking water bath (Grant Instruments, UK) for 15 min. before use.

### Measurement of heat tolerance of encapsulated spores

Initial studies with both *B. bassiana* and *A. lecanii* revealed spore populations of 3.9 × 10^7^ cells ml^−1^ and 8.2 × 10^7^ cells ml^−1^, respectively when grown on potato dextrose agar (PDA). In addition, the heat tolerance of spores of *B. bassiana* and *A. lecanii* was 50°C and 60°C, respectively. Therefore, the study progressed with the investigation into encapsulation of *A. lecanii* given the higher spore density and temperature tolerance. The spores of *A. lecanii* were collected in sterile deionized water (SDW) from the surface of culture growing for 7 days on malt extract agar. The volume of the remaining suspension was measured by weight in order to calculate the population of spores at the start of the experiment and then a sample was removed (100 µl) to determine the initial spore count by direct counts under the haemocytometer. The remaining spores were centrifuged at 6000 rpm for 5 min in a preweighed empty tube and the supernatant was discarded. The spore pellet was resuspended different suspensions, composed of varying proportions of Gelrite, cellulose, and standard Super Cel-fine (Diatomaceous Earth, calcined) that had been prewarmed in a water bath (45°C) (Table 1). These suspensions were prepared by heating and vigorously mixing to assist in resuspending the high Gelrite concentrations and then autoclaved (121°C, 15 min). The weight of the suspension was measured in order to calculate the total spore population (live and dead) and then the suspension was slowly dispensed through a 1 ml syringe into calcium chloride solution (0.1%), resulting in hardening of the Gelrite suspension and in the formation of beads. The remaining calcium chloride solution was removed by pipetting, the beads were washed in SDW and then freeze dried for 48 h. The beads were used immediately after freeze-drying. The weights of sterile Eppendorf tubes were measured and then filled with three beads each and the tubes were weighed again. Duplicate tubes containing the beads were filled with prewarmed autoclaved glycerol (200 µl) at 45°C, briefly centrifuged and incubated on a horizontal shaker (AccuTherm microtube shaking incubator, Labnet International Inc.) at the desired temperature, with shaking at 600 rpm for 2 min. The tubes were filled with glycerol only before immediate use. Glycerol was chosen to mimic conditions used during extrusion. The temperatures selected were 50°C, 60°C, 70°C, 80°C, 90°C, and 100°C. After shaking, SDW (800 µl) was added to each tube once cooled after being left at room temperature for 5 min, vortexed for 20 s, and then left at room temperature for 30 min. The beads were homogenized using sterile mini-pestles (Fisherbrand, Fisher Scientific) and then the contents were vortexed again for 1 min. The contents were serially diluted in SDW (900 µl) and then plated onto PDA spread plates. The plates were incubated for 1 week at 22°C and the colonies were counted.

**Table 1.** Composition of suspensions to prepare beads.

Encapsulated suspension	Gelrite (%)	Cellulose (%)	Cel-fine (%)
1	1.5	0.4	0.4
2	2	0.4	0.4
3	2	0.8	0.8
4	2	0.8	–
5	2	–	–

### Measurement of spore populations associated with single beads

The mass of single beads was determined by placing each bead (16 beads were assessed for each suspension) into preweighed tubes and measuring their weights. The beads (five) were resuspended in SDW (1 ml), left for 30 min, and then homogenized with sterile pellet pestles. The suspensions were serially diluted in SDW (0.9 ml) and then plated (100 µl) onto PDA. The plates were incubated for 1 week at 22°C and then colonies were counted.

### Measurement of heat tolerance of free spores

The heat tolerance of spores was determined by collecting spores in SDW from the surface of a plate growing for 1 week. The spores were centrifuged at 2414 × *g* for 5 min and the pellet was resuspended in prewarmed autoclaved SDW or glycerol (5 ml) at 40°C. The spores were vortexed for 1 min and aliquots were pipetted (200 µl) into sterile Eppendorf tubes, which were tested at different temperatures for 2 min in duplicate. The selected temperatures were 40°C, 50°C, 60°C, 70°C, 80°C, and 90°C for 2 min at 600 rpm. To each of the tubes, SDW (0.8 ml) was added once the contents were presumably at room temperature, vortexed, and plated onto PDA. Preparation of freeze-dried spores involved collecting material from the surface of a plate, concentrating by centrifuging as described previously, and placing aliquots (200 µl) into sterile cotton wool plugged Eppendorf tubes which were then frozen at −20°C with the caps sealed. The caps were opened, and the frozen tubes were placed into a sterile vessel plugged with cotton wool and freeze-dried for 48 h. The heat tolerance of the freeze-dried spores was determined in duplicate tubes as described previously.

### Determination of bead diameter using density measurement

A block of the encapsulated suspension 2 (Table 1) was prepared with calcium chloride solution (20 ml, 0.1%). The excess liquid was removed when the gel block had formed, the gel was frozen at −20°C and then freeze-dried for 48 h. The density of the freeze-dried block was determined using the density determination set for analytical balances (Kern & Sohn GmbH, Germany) by submersion under a wire support in methylated spirit where the density of methylated spirit was assumed to be 0.79 g cm^−3^. The density was used to calculate the diameter of the beads assuming they were spherical.

### Scanning electron microscopy of spores

Images of the surface of the beads and sliced images of the interior were captured using a scanning electron microscope (Hitachi TM4000 SEM) at magnifications ranging from 100x to 1000x.

### Insecticidal activity of encapsulated spores

Beads containing *A. lecanii* spores (20) were individually placed into small Petri dishes and each was moistened with sterile water (50 µl). The termites, *Macrotermes subhyalinus*, were sourced locally from the outskirts of Kampala, Uganda. One termite was placed into each Petri dish, which was kept in the dark at 25°C. The termites were checked after 1, 6, 12, 24, and 36 h and the number of surviving termites was recorded. Another set of beads without fungal spores (20) was examined under similar conditions.

## Results and discussion

### Formation of beads

The beads formed using 2% (w/v) Gelrite, 0.4% (w/v) cellulose, and 0.4% (w/v) Cel-fine appeared to have both characteristics of uniformity and the smallest mass (Fig. 1). Beads formed using lower concentrations of Gelrite [1.5% (w/v)] were irregular shaped and only a few beads could be formed using a much lower concentration of Gelrite [1% (w/v)]. The use of higher concentrations of calcium chloride during solidification of Gelrite may have improved the formation of stable beads. An attempt to form beads using higher concentrations of Gelrite [2.5% (w/v) and 3% (w/v)] was unsuccessful due to the increased viscosity. Beads formed with higher concentrations of cellulose and Cel-fine resulted in irregular shaped beads due to the high viscosity of the suspension. Beads formed using 2% Gelrite and 0.8% cellulose appeared somewhat spherical indicating that the addition of components to Gelrite was necessary to form spherical beads. It is anticipated that further improvements in bead formation could be achieved using alternative reagents such as calcium carbonate, causing the slow release of calcium ions resulting in firmer beads (Wang et al. 2023). In this study, it was revealed that 5% (w/v) sodium alginate beads formed with calcium chloride showed rapid hardening of the outer layer of the beads, whereas the inner part of the beads did not react with the reagent. The use of calcium carbonate overcame this problem.

### The effect of temperature on encapsulated spores

Spore populations associated with beads containing Cel-fine at 50°C appeared to be higher than beads without Cel-fine (Table 2), indicating that spores were most likely becoming embedded within pore spaces of Cel-fine thereby avoiding the immediate contact with the hot glycerol. About 100% of the spores used in the formation of beads containing Cel-fine before immersion into hot glycerol remained viable, whereas 70% of spores in beads containing only cellulose and 64% of spores containing only Gelrite remained viable (Fig. 1). While quantification of the spores in the beads at the start of the experiment was not replicated and is only indicative, another study also revealed that the addition of cellulose during the spray drying process resulted in the lowest survival of conidia (40%) compared with other additives (Felizatti et al. 2021). The spores had to withstand a temperature of at least 45°C for ~5 min during bead preparation. The addition of cellulose and/or Cel-fine to the beads was necessary for the spores to be resistant at a higher temperature of up to 80°C. The combination of the higher concentration of Gelrite along with mixtures of cellulose and Cel-fine showed the highest tolerance up to 100°C. The additional benefit of Cel-fine was shown in another study when searching for UV protectant of the spores because *A. lecanii* is highly susceptible to UV light causing a change in nutritional status from phototrophy to auxotrophy (Lee et al. 2006). This study revealed that montmorillonite, was most effective at 1% (w/v) concentration on UV-A and UV-C for more than 24 h, with only a 10% loss in germination efficiency and on UV-B for 6 h, indicating that the spores had become embedded within the montmorillonite and were protected from the UV rays. In our study, the beads formed using lower concentrations of cellulose and Cel-fine appeared to perform as well as those containing higher concentrations, despite the smaller masses of the individual beads and the closer promixity of the spores to the hot glycerol.

**Table 2.** Heat tolerance of beads prepared with different concentrations of Gelrite, cellulose, and Cel-fine.

	Temperature (°C)					
	50	60	70	80	90	100
1.5% Gelrite + 0.4% cellulose + 0.4 Cel-fine	6.04 ± 0.06 ^a^	6.10 ± 0.20 ^a^	6.20 ± 0.20 ^a^	5.35 ± 0.10 ^a^	0.00 ± 0.00 ^b^	0.00 ± 0.00 ^b^
2% Gelrite + 0.4% cellulose + 0.4 Cel-fine	6.49 ± 0.33 ^a^	6.23 ± 0.18 ^a^	6.06 ± 0.00 ^a^	5.67 ± 0.10 ^a^	5.58 ± 0.74 ^a^	4.70 ± 0.91 ^a^
2% Gelrite + 0.8% cellulose + 0.8 Cel-fine	6.45 ± 0.20 ^a^	5.34 ± 0.31 ^a^	5.49 ± 0.07 ^a^	5.35 ± 0.27 ^a^	2.33 ± 3.30 ^ab^	4.64 ± 0.03 ^ab^
2% Gelrite + 0.8% cellulose	4.87 ± 0.29 ^a^	3.66 ± 0.87 ^ab^	4.39 ± 0.26 ^ab^	4.28 ± 0.88 ^ab^	0.00 ± 0.00 ^b^	0.00 ± 0.00 ^b^
2% Gelrite	4.80 ± 0.77 ^a^	4.69 ± 0.04 ^a^	5.22 ± 1.61 ^a^	0.00 ± 0.00 ^b^	0.00 ± 0.00 ^b^	0.00 ± 0.00 ^b^

Values with same letters are not significantly different and values with different letters at *P* < .05.

Experimental procedures to test whether the spores could survive at the higher temperature of 150°C would require a different procedure because the current incubating shaker has a maximum temperature of 105°C. It would be difficult to establish vigorous agitation in an oil bath to mimic conditions obtained in the current study. Nevertheless, a linear extrapolation indicates that a few spores would survive although this quantity might be insufficient to infect termites.

### Bead mass and spore populations associated with individual beads

The masses of the beads were determined showing significant differences between the treatments (Fig. 1). Beads formed with 2% Gelrite, 0.4% cellulose, and 0.4% Cel-fine had the lowest masses (Fig. 1), whereas beads formed with a higher concentration of cellulose (0.8%) weighed most, presumably caused by swelling of cellulose. In contrast, the presence of 0.4% Cel-fine appeared to have no impact on bead mass compared with beads formed with only 2% Gelrite. The populations of spores associated with beads containing Cel-fine were significantly lower compared with beads without Cel-fine (Fig. 1). Populations associated with beads containing Cel-fine showed higher variances compared with other bead preparation containing Gelrite and cellulose or only Gelrite, which was evident by some beads containing Cel-fine possessing no fungal spores. This would seem to indicate a different mixing protocol would be required whereby the Cel-fine would be mixed with the spores immediately after collection. The low spore densities associated with the beads may not have a significant effect on insect populations individually, based on a previous study with *M. brunneum*, which reported that 10^5^ spores had a mortality rate of 13% on the fruit fly, *Ceratitis capitata*, compared with 7% with the uninfected controls (Yousef et al. 2014). Therefore, much higher populations than 10^8^ spores ml^−1^ would need to be achieved based on previous studies. For example, a high concentration of 1.1 × 10^10^ spores of *A. lecannii* per g dried carrier could be achieved when grown on corn flour, yeast extract, and potassium dihydrogen phosphate, using sugar bagasse as a solid substrate during solid state fermentation (Shi et al. 2009). One of the main factors in achieving high spore production was ensuring a low nitrogen content. In addition, there was little difference in spore concentrations obtained with different types of carbon and nitrogen sources and the cost of the ingredients became the important factor in selecting the raw materials. An evaluation of other substrate materials for solid substrate fermentation indicated that spore yields on sugarcane bagasse were higher compared with those grown on wheat straw, corncob, and activated carbon and occurred more quickly compared with polyurethane foam (Xu et al. 2011). Scanning electron microscopy (SEM) revealed a higher proportion of pores spaces in polyurethane foam enabling the development of more aerial mycelium compared with sugarcane bagasse. However, the higher nutritional content associated with sugarcane bagasse resulted in more vegetative mycelium and in turn higher spore densities.

The construction of the beads was based on materials that would lower thermal conductivity which was determined for cellulose as 0.076 W mh^−1^ at 25°C (Dombek et al. 2020) and Cel-fine, which has a similar composition to clay having a low thermal conductivity property with a median of 1.4 W mh^−1^ (Zhu et al. 2018). The thermal conductivity of cellulose was found to increase to 0.121 W mh^−1^ when heated at 100°C. The beads were immersed in glycerol to mimic one of the ingredients used during the extrusion process, where the thermal conductivity of glycerol was determined to be 0.29 W mh^−1^, which is lower than water at 0.56 W mH^−1^ (Sharifpur et al. 2017). However, a previous study has shown that increasing glycerol concentrations led to an increase in osmotic potential, thereby reducing the ability of *A. lecanii* spores to germinate and grow (Chandler et al. 1994). Some of these strains did appear to show different tolerances to osmotic potentials to glycerol.

### Comparison between encapsulated spores and free spores

**Figure 2.**
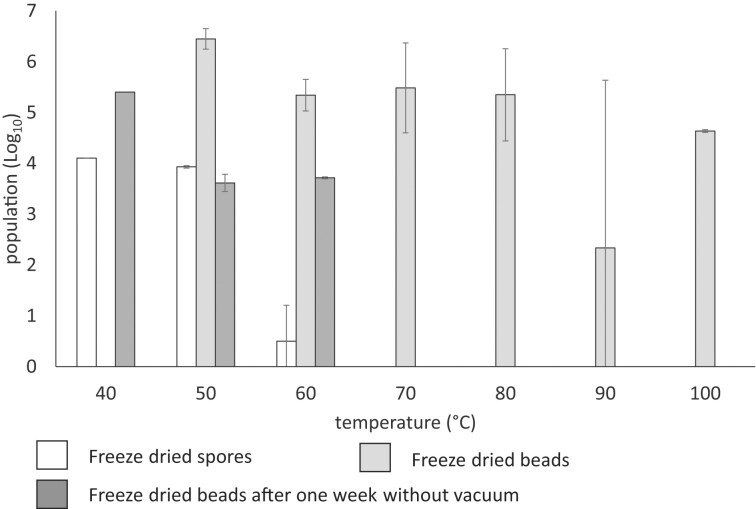
Heat tolerance of spores resuspended in SDW, and spores encapsulated in beads containing 2% Gelrite, 0.4% cellulose, and 0.4% Cel-fine immediately after freeze-drying and 1 week left at ambient temperature after being freeze-dried.

It was evident that the encapsulated spores showed a clear improvement in heat tolerance compared with free spores (Fig. 2). Furthermore, free spores immersed in glycerol showed no ability to germinate at temperatures ranging from 25°C to 100°C, supporting the results obtained in another study where glycerol caused osmotic shock (Chandler et al. 1994).

### Comparison of immediate and delayed use of encapsulated spores

It was also determined that beads maintained at ambient temperature for a week showed a decrease in thermal tolerance of the encapsulated spores, compared with those tested immediately after freeze-drying, presumably due to the absorption water vapor (Fig. 2). Therefore, future applications of these beads during extrusion would require their immediate use after freeze drying. Strategies to improve the heat tolerance of the encapsulated spores could be achieved by the incorporation of soya oil to the suspension before preparation of the beads, in order to increase their hydrophobic surface properties (Felizatti et al. 2021). Furthermore, the beads could be prepared with sodium carbonate, which would slow down the rate of bead formation (Wang et al. 2023), perhaps resulting in more complete formation of hardened material throughout the beads. Furthermore, the freeze-beads could be coated with a chitosan layer that may act as a more permanent barrier to the diffusion of hot glycerol entering the pore spaces of the beads (Willaert and Baron 1996). Finally, the extrusion temperature could be reduced to 100°C if glycerol was replaced with water, which has been shown to be effective with rice flour (Zambrano et al. 2022) although films produced with water have been found to be more brittle. However, thermal heat conduction in water is higher that glycerol, which may result in a lower spore temperature tolerance.

### Density of beads

The standard density of a block of 2% Gelrite + 0.4% cellulose + 0.4% Cel-fine was measured because spores showed optimal survival at the higher temperatures. Furthermore, these beads appeared almost spherical, which would lead to accurate calculations of diameters. The density was determined to be 0.1674 g cm^−3^ and calculation of the bead diameter was determined to be 2.85 ± 0.06 mm assuming the beads were spherical. These values appeared to be similar to the apparent bead size shown under SEM showing diameters were larger than 2 mm (Fig. 1).

### Microscopy of encapsulated spores within the beads

**Figure 3.**
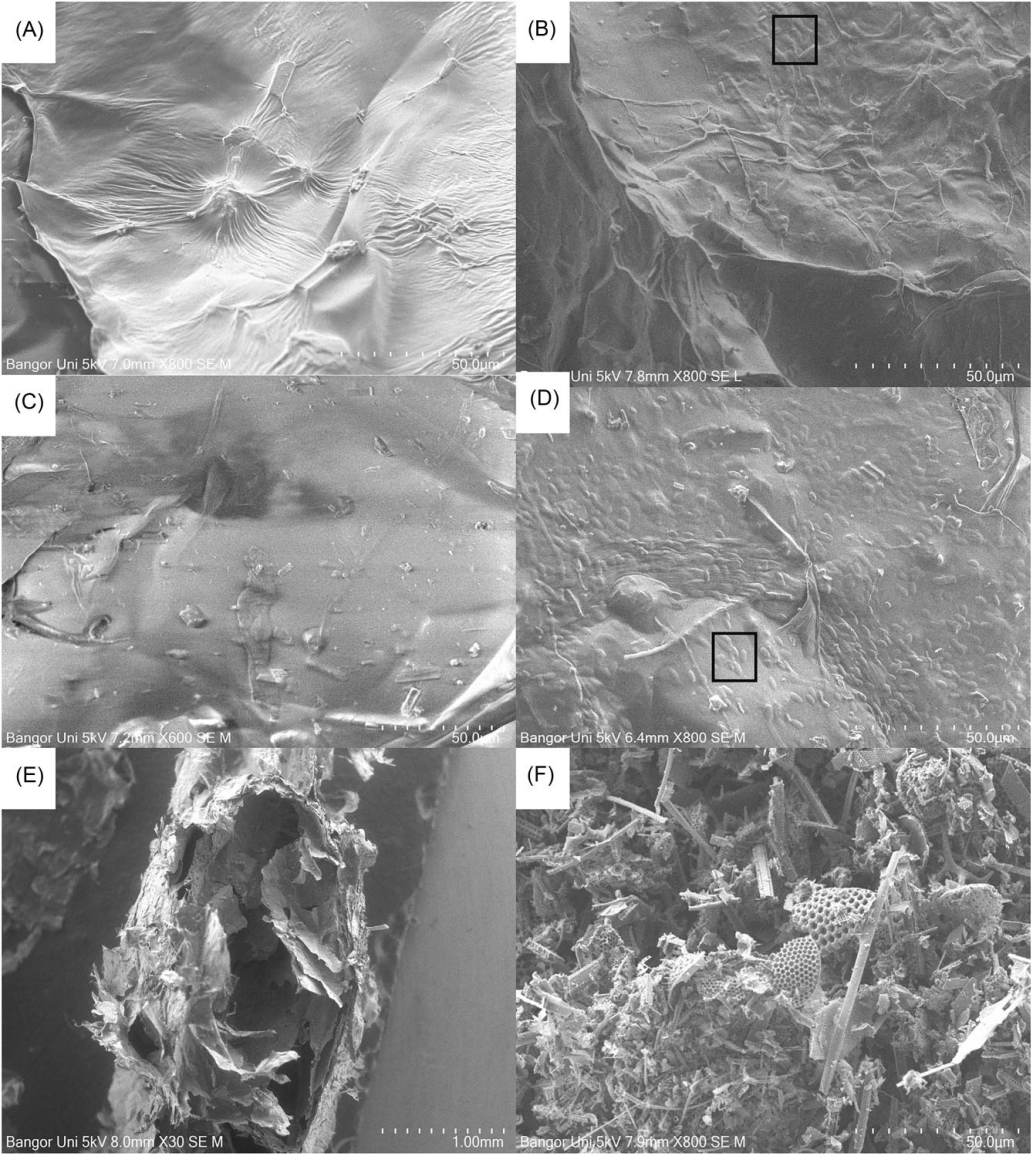
Bead surface without fungus (A) and with fungus (B). Bead interior without fungus (C) and with fungus (D). Cross-section of bead (E) and Cel-fine particles (F). Ellipsoid cells inset within box of images of B and D.

SEM revealed more spores were present within the interior of the 2% Gelrite + 0.4% cellulose + 0.4% Cel-fine beads compared with the exterior of the beads (Fig. 3). The structure of the bead revealed layers, perhaps indicating that only the external layer had solidified and had separated into layers during freeze drying. Gelrite has been described to form a double helix in the presence of monovalent and divalent cations, which combine to create layers (Banik et al. 2000). Pressumably, spores closest to the bead surface would have been destroyed during heating whereas spores located within the center of the beads would be protected by the many layers of the bead. During the extrusion process, it would be expected that the beads would be transformed into flattened spheres, leading to spores in the middle of the beads being closer to the surface, resulting in a lower temperature tolerances of these spores.

### Effect of encapsulated spores on termites

**Figure 4.**
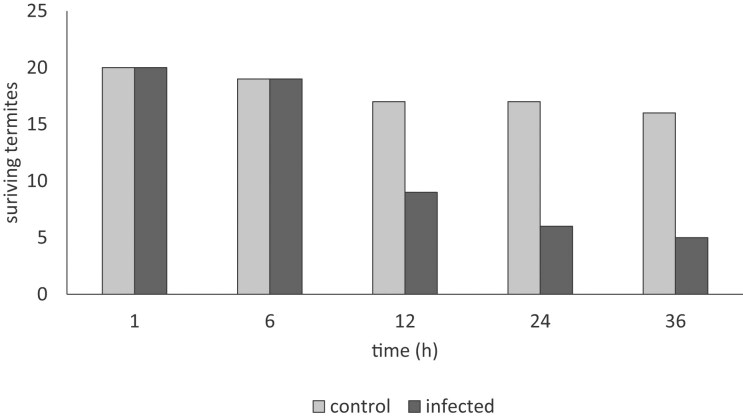
Survival of termites with control beads containing no fungal spores and infected beads containing fungal spores.

An initial study was performed with waxworms, which seemed to indicate that *A. lecanii* could cause infection through ingestion rather than penetration through the exoskeleton (data not shown). The beads containing *A. lecanii* spores were examined with termites, *M. subhyalinus*, appearing to show a decline in the remaining surviving termites compared with the control beads which contained no spores (Fig. 4). It is uncertain whether the spores, caused infection in the termites leading to death or whether the termites were avoiding the beads because termites are known to avoid potentially infectious spores. The termites would become susceptible to starvation if the termites were avoiding consuming the beads because there was no other food source available. Further work would need to be performed in replicates and the presence of fungal infection would have to be assessed by molecular techniques.

Only one study examined the infectivity of one species of *A. lecanii* against termites, but this was an ineffective strategy (Khan et al. 1991). However, this same study also reported major differences of infectivity of different strains of *B. bassiana* against different species of termites to reveal that one species was more dominant. Such differences in infectivity may also occur with different strains of *A. lecanii*.

## Conclusions

These results show the heat tolerance of *A. lecanii* spores could be improved by encapsulation into 2% Gelrite beads containing 0.4% cellulose and 0.4% Cel-fine. However, the current method of extrusion would involve a temperature of 150°C for 2 min, which would still require further development to raise the temperature tolerance of the encapsulated spores. Alternatively, a different extrusion process could be developed which involves a lower temperature around 100°C using water instead of glycerol. Consequently, further analysis would be required to evaluate the temperature tolerance of the encapsulated beads in water. Nevertheless, the results indicate that *A. lecanii* encapsulated beads may be effective against termites through infection or as a deterrent.
